# Thin-film implants for bioelectronic medicine

**DOI:** 10.1557/s43577-024-00786-7

**Published:** 2024-09-26

**Authors:** Poppy Oldroyd, Salim El Hadwe, Damiano G. Barone, George G. Malliaras

**Affiliations:** 1 Electrical Engineering Division, Department of Engineering, University of Cambridge, Cambridge, UK; 2 Department of Clinical Neurosciences, University of Cambridge, Cambridge, UK

**Keywords:** Bioelectronic, Thin film, Biomedical, Devices

## Abstract

*This article is based on the MRS Mid-Career Researcher Award “for outstanding contributions to the fundamentals and development of organic electronic materials and their application in biology and medicine” presentation given by George G. Malliaras, University of Cambridge, at the 2023 MRS Spring Meeting in San Francisco, Calif.*

Bioelectronic medicine offers a revolutionary approach to treating disease by stimulating the body with electricity. While current devices show safety and efficacy, limitations, including bulkiness, invasiveness, and scalability, hinder their wider application. Thin-film implants promise to overcome these limitations. Made using microfabrication technologies, these implants conform better to neural tissues, reduce tissue damage and foreign body response, and provide high-density, multimodal interfaces with the body. This article explores how thin-film implants using organic materials and novel designs may contribute to disease management, intraoperative monitoring, and brain mapping applications. Additionally, the technical challenges to be addressed for this technology to succeed are discussed.

**Graphical abstract:**

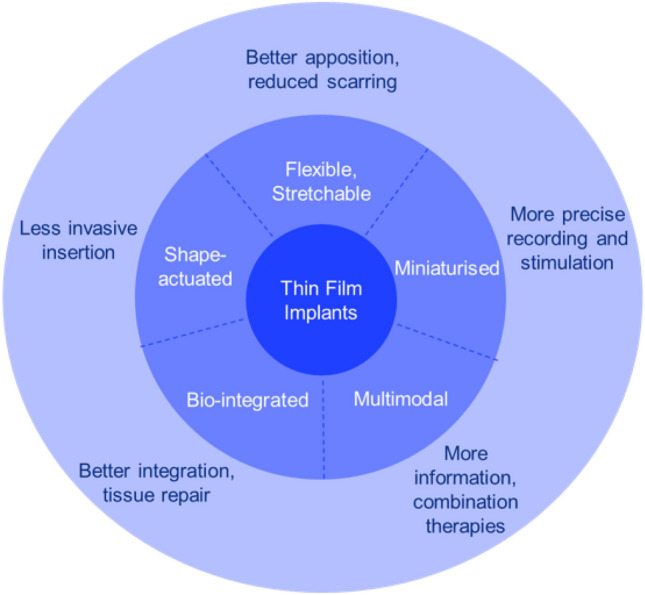

## Introduction

Drug administration and surgical intervention have long been the cornerstones of medicine, aiming to restore health through chemical or “mechanical” ways. Bioelectronic medicine is an emerging way to practice medicine by harnessing the body’s electrical signaling pathways.^1^ It uses implantable electronic medical devices (IEMDs) to deliver electrical stimulation to targeted tissues, thereby modulating neural activity to treat diseases or restore physiological function. This approach offers a promising avenue to address limitations associated with conventional therapies, such as poor efficacy, off-target effects, and high costs.^2^ The origins of bioelectronic medicine can be traced back to the eighteenth century, with Luigi Galvani’s experiments on electricity’s effect on living tissues. The 1958 development of the first fully implantable pacemaker marked a significant turning point. Advancements in materials, circuits, and packaging have enabled the development of increasingly sophisticated IEMDs for stimulation and recording at different parts of the nervous system, targeting an expanding array of indications. This rapid progress fuels market growth, with projections estimating a global market size of USD $16–60 billion within the next decade.^3^

IEMDs are used in both short-term (acute/subacute) settings, such as intracranial monitoring for epilepsy or trauma,^4^ and long-term (chronic) settings, such as cardiac pacemakers for treating arrhythmias.^5^ They provide real-time data, aiding diagnosis, management, and treatment, revolutionizing patient care. Such devices have established themselves as a valuable tool for treating neurological disorders. Deep brain stimulation (DBS) is a well-established therapy for movement disorders.^6^ Vagus nerve stimulation (VNS) offers a treatment option for epilepsy,^7^ and spinal-cord stimulation (SCS) is a sustainable solution for chronic pain management.^8^ Cochlear implants have shown success in treating hearing loss by directly stimulating the auditory nerve.^9^ The potential of neurotechnology extends beyond these established applications. Clinical trials are underway for using these devices to treat various conditions under FDA investigational use, including focal and generalized epilepsy, pain, and Alzheimer’s disease.^10^ The field of neuropsychiatry is also embracing bioelectronic medicine. Studies suggest that stimulation techniques may hold therapeutic value for obsessive–compulsive disorder (OCD) and depression.^11,12^ Brain-computer interfaces (BCIs) are actively being explored to restore movement in paralyzed individuals.^13,14^ Results from ongoing clinical trials showcase its efficacy in treating chronic medical conditions (e.g., rheumatoid arthritis^15^) and metabolic dysregulation (e.g., diabetes^16^).

**Figure 1:**
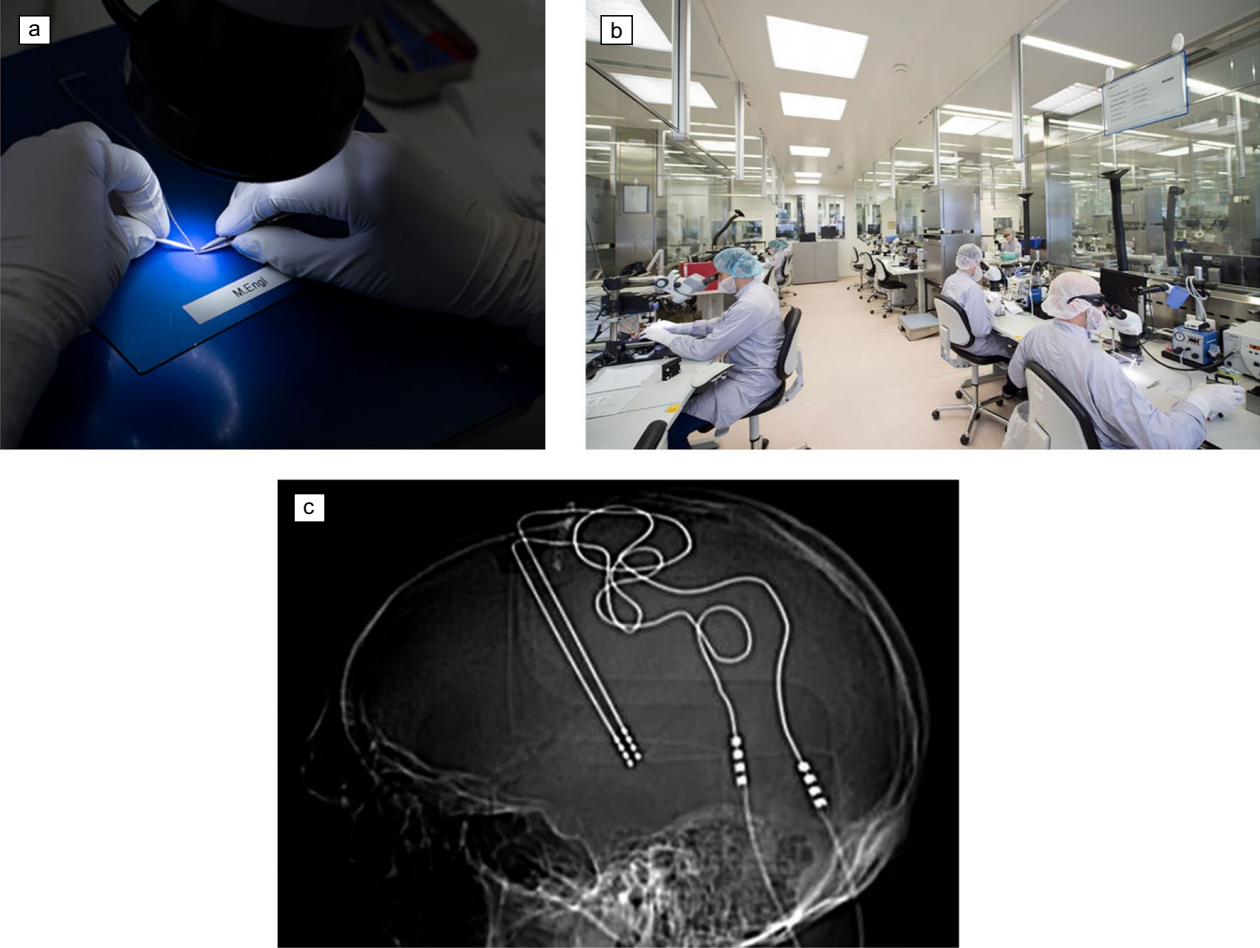
(a, b) Fabrication of current implants for bioelectronic medicine. The process involves a manual assembly line. Reprinted with permission from Reference 19. © 2016 MED-L. (c) Postoperative lateral x-ray shows deep brain stimulation leads implanted in the left and right subcallosal cingulate region. Reprinted with permission from Reference 26. © 2019 *Proceedings of the National Academy of Sciences*.

Despite significant advances in neurotechnology, the manufacturing process for many IEMDs remains surprisingly manual (**Figure** **1**a–b), with techniques that have seen little evolution since the first cochlear implants were developed in the late 1960s.^17,18^ Current IMEDs typically feature an electrode array with a small number of millimeter-sized Pt/Ir electrodes connected to an implantable pulse generator in which the electronic components and battery are housed within a hermetic casing, often made of titanium.^19^ Medtronic’s Activa RC neurostimulator for DBS, with two electrode arrays for bilateral implantation, is shown in Figure 1c. Cochlear’s Nucleus CI512 cochlear implant utilizes 22 platinum–iridium (Pt/Ir) electrodes encased within a thick layer of silicone.^20^ Boston Scientific’s percutaneous leads for spinal-cord stimulation employ a similar structure,^21^ with 16 electrodes on a linear array with a diameter of 1.3 mm. The bulkiness of current IEMDs makes them rather invasive, as illustrated by the DBS system in Figure 1d. It also results in high-electrode migration rates, leading to a loss of efficacy.^22^ The materials used have dramatically different properties than living tissue, leading to a pronounced foreign body response (FBR) that also compromises efficacy.^23^ Moreover, the small number of relatively large electrodes leads to poor treatment specificity and off-target effects.^24^ These characteristics prohibit bioelectronic medicine from reaching its full potential.

## Thin-film implants

**Figure 2:**
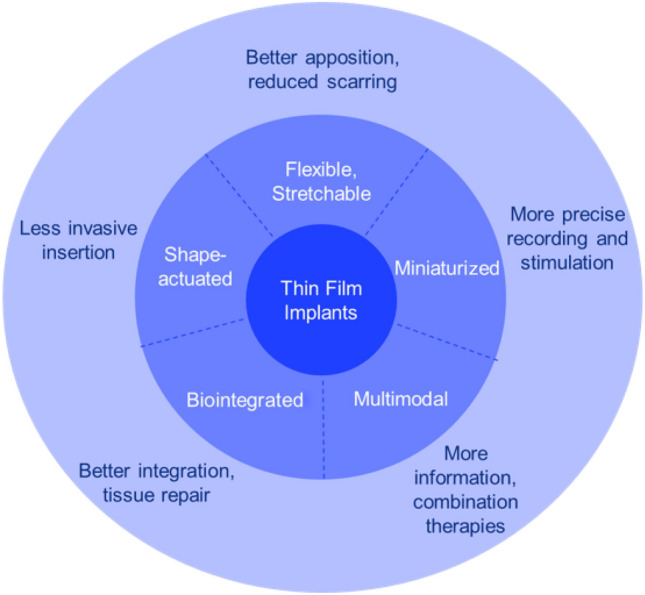
Value proposition for thin-film devices in bioelectronic medicine. The middle darker blue circle highlights the recent achievements of thin-film bioelectronics, and the lighter blue outer circle highlights the benefits these new devices offer compared to traditional bioelectronic devices.

The issues facing current IEMDs have spurred the development of implants using techniques developed for the microelectronics industry, including thin-film deposition, photolithography, and etching.^27,28^ This allows electrode arrays to be fabricated to a smaller size (typical thickness of a few micrometers compared to a few millimeters in current electrode arrays), meaning they significantly attenuate the foreign body reaction.^27,28^ In the form of two-dimensional (2D) microelectrode arrays (MEAs), they feature 100 s or 1000 s of micrometer-sized electrodes (compared to 10 s of millimeter-sized electrodes in current implants) and conform to large areas of tissue, providing exceptionally high resolution.^29^ As a result of these desirable properties, they have gained attention over the past few decades for their use in neuromodulation,^30–36^ addressing the growing need for minimally invasive,^37^ highly selective devices, enabling precise stimulation and recording of the nervous system.^38^ Recent literature provides powerful demonstrations of thin-film implants that are ultra-conformal,^39^ stretchable,^40^ multiplexed,^41^ and bioresorbable.^42^ In** Figure** 2, we highlight some of the features of thin-film implants (middle circle), and the benefits of bioelectronic medicine (outer circle) that derive from these, with examples discussed below.

### Flexible and stretchable

The inherent rigidity of bioelectronic devices, particularly those composed of silicon (Young’s modulus around 10 GPa), creates a significant mechanical mismatch when implanted in soft brain tissue (neuronal Young’s modulus: 0.02–0.04 kPa, glial cells: 1–10 kPa).^43^ This mismatch is a major contributor to the FBR, resulting in a chronic inflammatory reaction that disrupts brain homeostasis and leads to scarring.^44,45^ Beyond mechanical flexibility, factors such as implant shape, size, and surface texture can influence the FBR, emphasizing the importance of design for long-term integration.^46,47^ Flexible and stretchable thin-film electrodes are promising solutions to these challenges. Unlike their rigid counterparts, these soft devices reduce the mechanical mismatch and conform to the tissue, minimizing micromotion and potentially mitigating the FBR. Early work employed sacrificial layers such as dissolvable silk fibroin films to enable the surgical handling of ultrathin, conformal electrodes.^39^ Mounting these devices on tissue and subsequently dissolving the silk initiates a spontaneous wrapping process driven by capillary forces at the tissue–device interface. When implanted in a feline brain, the ultra-conformable electrodes offered a 70% increase in the signal-to-noise ratio, with no evidence of immune response.^39^

**Figure 3:**
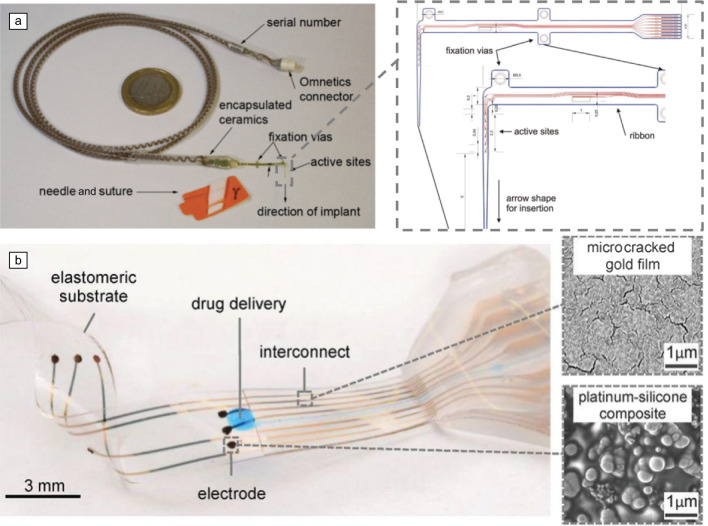
Two examples of assembled flexible and stretchable thin-film devices. (a) The transversal intrafascicular multichannel electrode-3 electrode for interfacing with peripheral nerves. Reprinted with permission from Reference 50. © 2019 Taylor & Francis. (b) E-dura: a flexible and stretchable thin-film device for interfacing with tissue. Reprinted with permission from Reference 40. © 2015 AAAS.

The flexibility and conformability of thin-film electrodes enhance access to previously challenging areas of neural tissue. Unlike bulky electrodes that are not suitable for interfacing with the delicate peripheral nervous system, thin films enable high-quality sub-neural resolution recordings (as shown in^48,49^). A prime example is the transversal intrafascicular multichannel electrode (TIME),^50^ which leverages this advantage to achieve both nerve cross-sectional coverage and local selectivity in a single device (**Figure** **3**a). In this work, thin layers of polyimide (PI) and metal produce a flexible device only 11-µm thick, which, when implanted in a rat sciatic nerve, remained functional for more than two months with minimal damage.^51^ Recent research has introduced silicone-based soft cuff electrodes for selective nerve stimulation. These adaptable designs ensure precise, pressure-free contact and minimize tissue damage.^52^ Additionally, ultra-conformable cuff implants have been developed that can record and stimulate peripheral nerves with fascicle-specific resolution, potentially revolutionizing treatments for nerve injuries.^48^ Moreover, dynamic tissues such as those in the enteric nervous system are now more accessible as thin-film implants have been shown to provide effective modulation of gut and intestine functions, offering relief for conditions such as irritable bowel syndrome, gastroparesis, and inflammatory bowel disease.^53,54^

Another promising, previously hard-to-reach target is the spinal cord. The “electronic dura” device, designed to mimic the elasticity of the dura mater (the spinal cord’s protective membrane) using an elastomeric substrate, has shown promise for long-term biointegration following sub-dural implantation. This design approach reportedly minimizes the foreign body response, offering a potential solution for chronic spinal-cord interfacing (Figure 3b).^40^

Another approach utilized PI/Au/PI stacks to create electrodes targeting the ventral portion of the spinal cord, closer to motor circuits. The flexibility of these electrodes allows for implantation within the ventral epidural space. Additionally, the design enables wireless control, eliminating the need for percutaneous wires. This approach offers the ability to target areas previously inaccessible with traditional methods.^55^ Furthermore, the inherent flexibility allowed similar electrodes to conform to the entire spinal-cord circumference.^56^ A “360 device” fabricated from parylene C and conducting polymer electrodes demonstrated the ability to record and stimulate multiple motor and sensory signals around the spinal cord in anesthetized rats as well as be used in a closed-loop system to bridge an acute complete spinal-cord injury using dual circumferential devices.^56^

### Miniaturized

**Figure 4:**
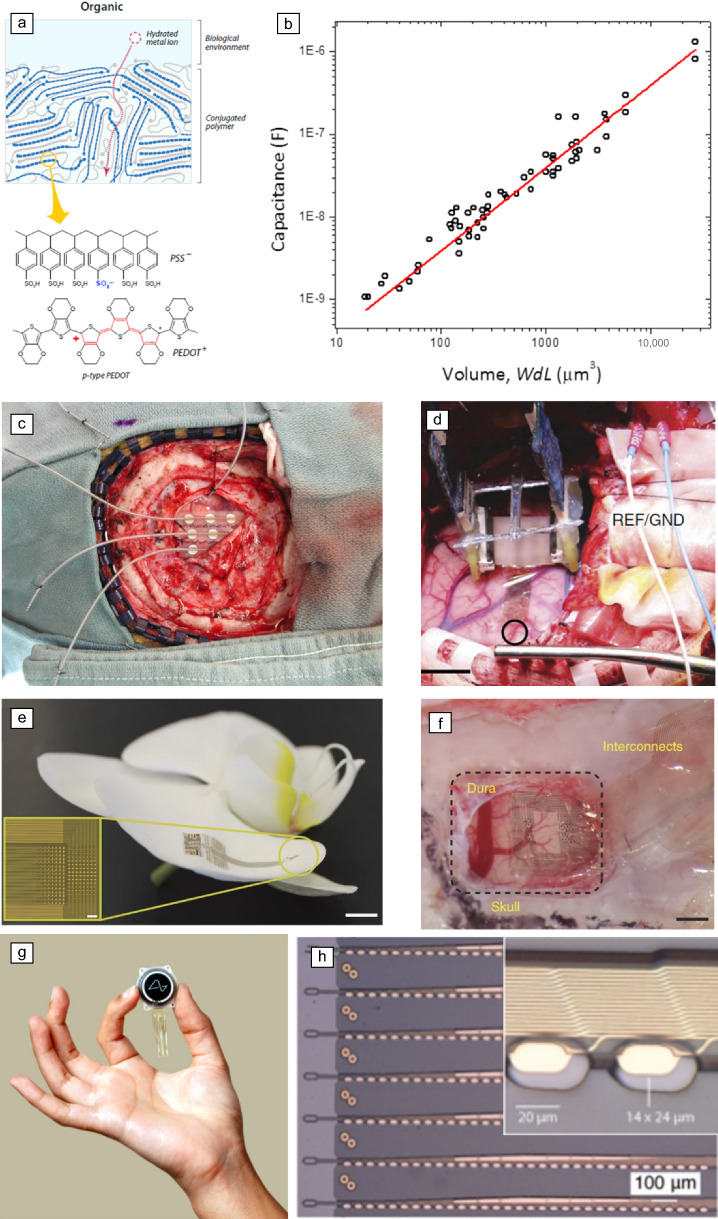
Examples of miniaturized thin-film devices. (a) Schematic showing PEDOT:PSS/electrolyte interface and structure. Reprinted with permission from Reference 61. © 2013 American Chemical Society. (b) Volumetric capacitance of PEDOT:PSS. Reprinted with permission from Reference 62. © 2015 AAAS. (c) Pt/Ir contacts, 5-mm diameters with nine electrodes. (d) PEDOT:PSS thin-film electrodes, 10-µm diameters with 256 electrodes. Reprinted with permission from Reference 29. © 2015 Springer Nature. (e) NeuroGrid: flexible device conforms to orchid petal. Scale bar = 5 mm. Inset: High-density electrodes (256) on NeuroGrid. Scale bar = 100 μm. (f) Brain Interface: NeuroGrid integrates with rat somatosensory cortex. Scale bar = 1 mm. Reprinted with permission from Reference 29. © 2015 Springer Nature. (g, h) Neuralink’s thin-film brain implant has more than 1000 channels. Reprinted with permission from Reference 63. © 2019 JMIR Publications. REF, reference, GND, ground.

Miniaturizing neural implants requires overcoming the limitations of traditional metal electrodes. A decrease in electrode area is accompanied by an increase in impedance and a reduction in charge injection capacity, and this hinders functionality at smaller scales. A significant breakthrough in overcoming these limitations is the use of organic mixed ionic–electronic conductors (OMIECs).^57^ These novel materials enable thin, flexible, and conformable electrodes with superior electrochemical properties. Unlike metals, OMIECs boast low impedance and high charge injection capacity due to their combined ionic and electronic conductivity.^58,59^ This enables high-resolution recording and safe neural stimulation from microelectrodes. One prominent example is the conducting polymer poly(3,4-ethylenedioxythiophene) poly(styrene sulfonate) (PEDOT:PSS) (**Figure** **4**a), which exhibits high volumetric capacitance, leading to two orders of magnitude lower impedance than conventional Pt/Ir electrodes of the same area (Figure 4b). This enables a significant reduction in electrode size, enabling the development of high-density microelectrode arrays unseen with traditional materials (Figure 4c–d). For instance, the NeuroGrid uses PEDOT:PSS to create arrays with electrodes with 10 × 10 µm^2^ surface area and 30-µm interelectrode spacing (Figure 4e–f).^29^ These thin (4 µm) and ultra-conformable devices enable researchers to capture single-neuron neural activity from the cortex of rodents and humans, a feat previously requiring penetrating electrodes.

It is important to acknowledge that advancements beyond OMIECs are being explored. The “Neural Web” device exemplifies this with its open lattice structure that provides conformal and tight contact to the surface of the brain and enables high-density recordings.^60^ The “Neuralink” device exploits the use of iridium oxide (IrOx) to lower the impedance of their high-density electrodes, enabling them to produce devices with 3072 channels for high-density brain recordings (Figure 4g–h).^63^ Ongoing research into low impedance, high charge storage, and injection capacity materials holds promise for further advancements in neural interface technology.

### Multimodal

**Figure 5:**
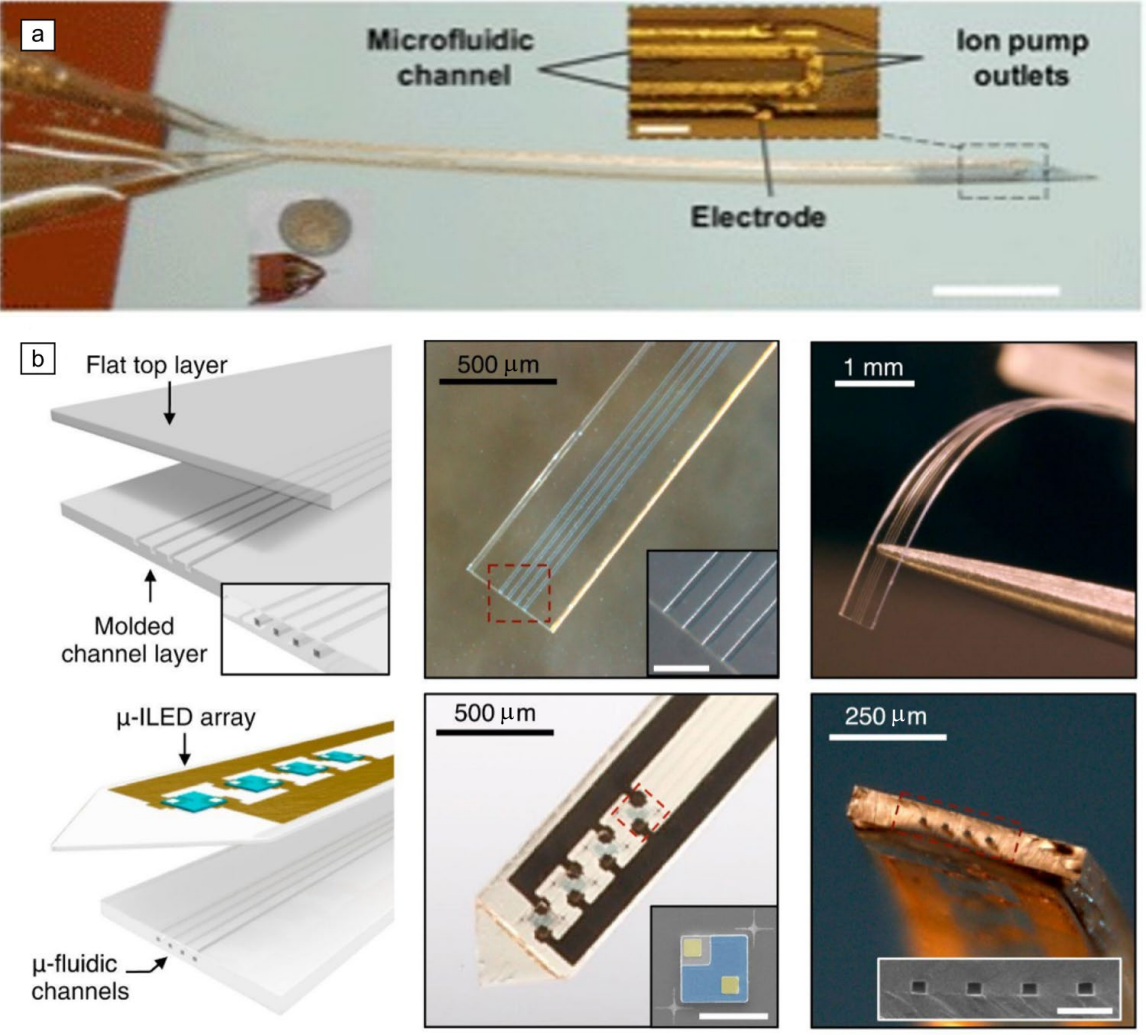
Examples of multimodal devices. (a) Example of a microfluidic drug delivery device integrated into a thin-film electrode. Inset scale bar: 100 µm; Main image scale bar = 1 mm. Reprinted with permission from Reference 66. © 2018 AAAS. (b) Example of a microfluidic device for drug delivery combined with light-emitting diodes (LEDs) to perform optical stimulation of specific neuronal populations. Row 1, Image 2: Inset scale bar: 100 µm. Row 2, Image 2: Inset scale bar: 100 µm. Row 2, Image 3: Inset scale bar: 50 µm. Reprinted with permission from Reference 68. © 2015 Cell Press. ILED, inorganic light-emitting diode.

While electrical recording has been the mainstay for studying neural activity, it offers a limited window into the complex biological processes within the brain. Multimodal thin-film probes address this limitation by integrating various sensing and stimulation modalities (electrical, chemical, and/or optical) to provide a more comprehensive interfacing with neural tissue. This opens exciting avenues for developing novel therapeutic strategies for a variety of neurological diseases. For example, traditional drug delivery for neurological diseases such as epilepsy often suffers from limitations, such as long regimens, narrow therapeutic windows, and complex schedules, hindering patient compliance and efficacy, particularly for unstable drugs.^64^ Integrating drug delivery into thin-film electrodes holds promise for overcoming these limitations. These devices can incorporate microfluidic channels for targeted drug delivery alongside microelectrodes for electrical recording, enabling combined therapeutic intervention and real-time neural monitoring (**Figure** **5**a).^40^ Research continues to explore novel technologies such as micropumps and miniaturized foldable devices for minimally invasive, multimodal drug delivery.^65^ These advancements hold promise for treating various conditions, as demonstrated in epilepsy models.^66^ Additionally, sensor-based systems can be integrated to monitor biological parameters and dynamically adjust drug release as needed, creating a more responsive therapeutic approach.^67^

Recent advancements in thin-film implants integrated micro-LEDs directly into the electrode design (Figure 5b), enabling targeted optogenetic stimulation of specific neuronal populations with precise wavelengths of light.^68^ This combined approach has improved our understanding of brain function, particularly in domains such as the link between brain activity and the mechanism of deep brain stimulation for Parkinson’s disease.^69^ Techniques such as simultaneous fluorescence imaging and modulation^70^ further exemplify the expanding possibilities of multimodal probes for *in vivo* monitoring and neurostimulation.

**Figure 6:**
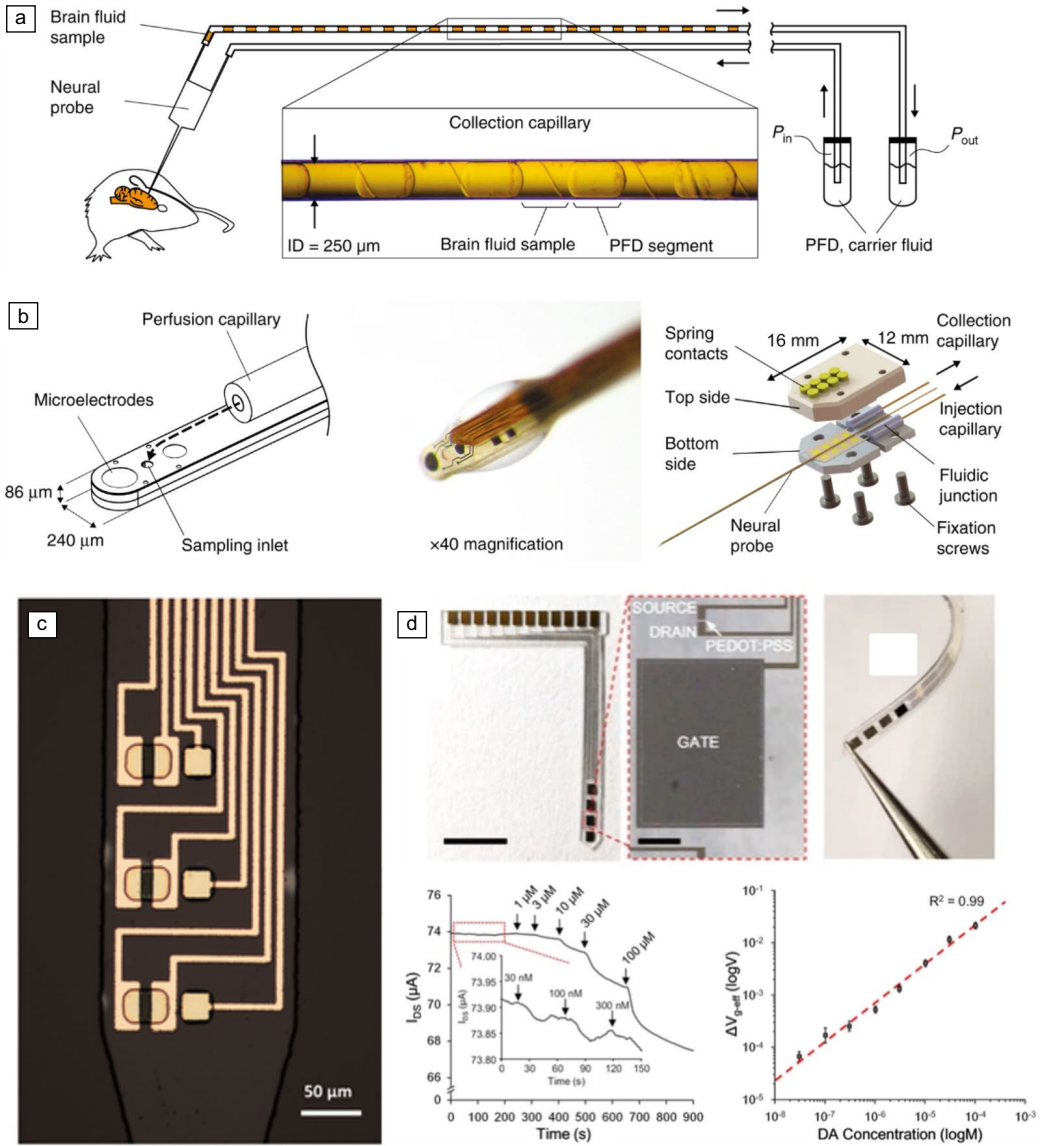
Biosensing thin-film devices. (a, b) Schematics of a thin-film neural probe with a capillary integrated into the lead wire for the collection of biological samples for further chemical analysis. Reprinted with permission from Reference 72. © 2017 Springer Nature. (c) Example of a thin-film organic electrochemical transistor (OECT) device for biosensing. Reprinted with permission from Reference 82. © 2020 MDPI. (d) Example of another OECT-based device, with examples of sensing different concentrations of dopamine. Row 1: Left image: Scale bar: 5 mm; Middle image scale bar: 200 µm. Reprinted with permission from Reference 83. © 2020 Wiley.

Thin-film devices can be further enhanced by incorporating chemical sensing capabilities.^71^ Microelectrodes are ideal for biosensing due to their fast response times, high signal-to-noise ratios, and ability to function in low-conductivity environments.^72^ One approach involves a droplet collection system at the probe tip for later chemical analysis (**Figure** **6**a–b).^73^ Organic electrochemical transistors (OECTs) integrated into these implants have shown success in sensing neurotransmitters such as dopamine and serotonin, contributing to the understanding and treatment of neurological disorders (Figure 6c).^74–76^ These OECTs can also detect biomarkers for diabetes (glucose),^77^ monitor acid–base balance (pH),^78^ and measure ion concentrations (sodium, potassium, calcium),^79^ providing real-time data for personalized medicine (Figure 6d). Biorecognition elements, such as enzymes or specific molecules, can be directly applied to electrodes or transistors for even more targeted sensing (e.g., DNA or protein detection).^80,81^

**Figure 7:**
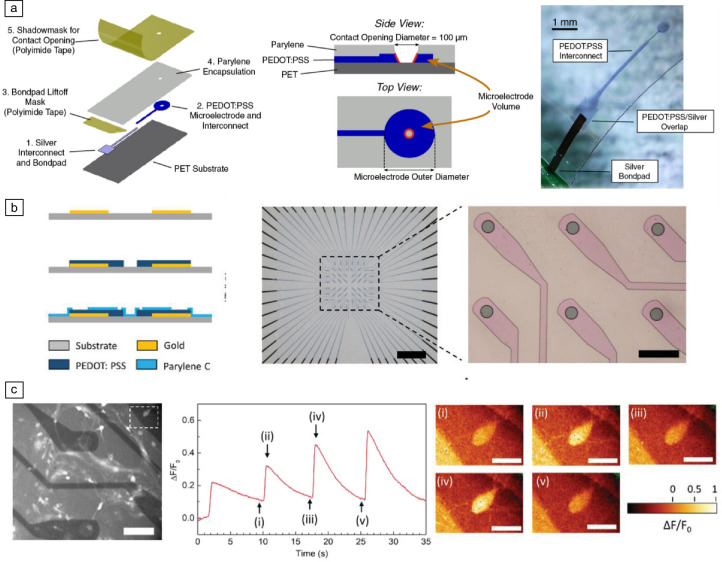
Transparent devices for simultaneous imaging and electrophysiology. (a) Schematic and realization of a fully PEDOT:PSS-based device for multimodal neural electrodes. Reprinted with permission from Reference 84. © 2021 Wiley. (b) Schematic and optical micrographs of another fully PEDOT:PSS thin-film device. Middle image scale bar: 1 cm; Right image scale bar: 100 µm. Reprinted with permission from Reference 85. © 2016 Frontiers Media. (c) Application of a PEDOT:PSS device for calcium imaging and subsequent fluorescence analysis. Left image scale bar: 50 µm; Right image scale bars: 5 µm. Reprinted with permission from Reference 85. © 2016 Frontiers Media.

Traditional thin-film devices are often opaque due to metal layers, hindering the use of advanced imaging techniques alongside the electrodes. Transparent microelectrodes fabricated from conducting polymers such as PEDOT:PSS are being developed to facilitate simultaneous optical and electrophysiological interfacing for neuroscience research (**Figure** **7**a).^84^ These transparent devices offer compatibility with calcium imaging and super-resolution optical microscopy techniques (Figure 7b–c),^85^ overcoming the limitations of conventional metal-based electrodes.

### Shape actuated

**Figure 8:**
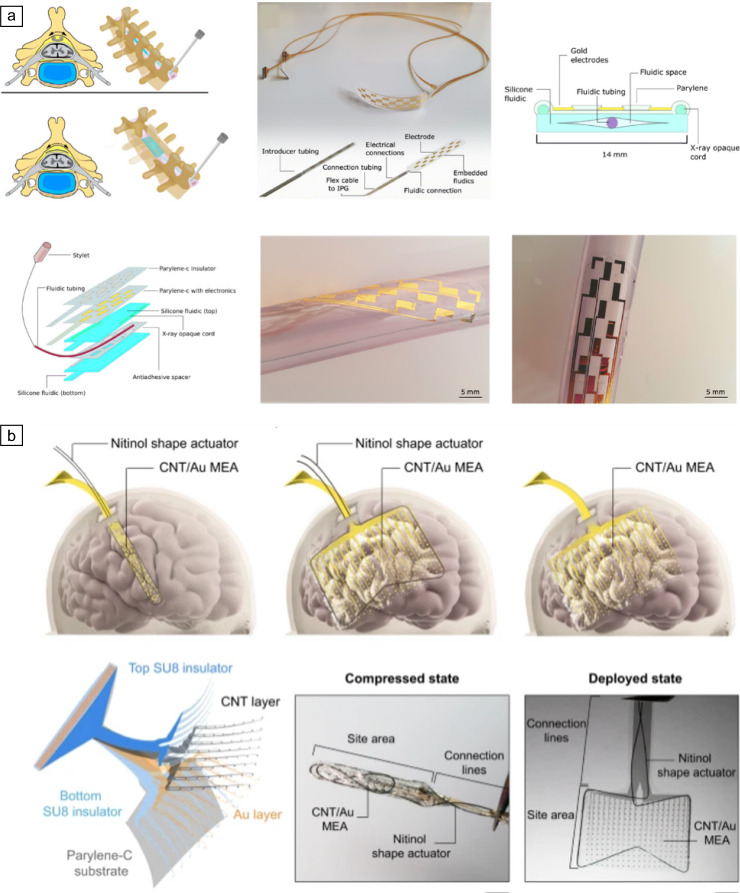
Shape-actuated devices. (a) Design and fabrication aspects of a minimally invasive spinal-cord device. Reprinted with permission from Reference 37. © 2021 AAAS. (b) Visualization of a nitinol-based minimally invasive brain implant in both the compressed and deployed state. Row 2: Middle image scale bar: 5 mm; Right image scale bar: 5 mm. Reprinted with permission from Reference 88. © 2015 Cell Press. IPG, implantable pulse generator, CNT, carbon nanotube, MEA, microelectrode array.

Conventional rigid bioelectronic implants come with a significant drawback: implantation damage. These devices often cause tissue tearing, bleeding, and inflammation during and after insertion due to their rigidity and surgical invasiveness necessary for implantation.^86^ Researchers are exploring minimally invasive implantation techniques with thin-film devices to address this. These aim to minimize tissue disruption and promote long-term biocompatibility following insertion.^45^ Minimally invasive implants leverage shape actuation to reduce their initial implantation footprint. Pneumatic actuation is a common technique, exemplified by soft electrocorticography (ECoG) systems made from elastomeric substrates.^87^ These systems deploy a miniature robotic actuator through a burr hole. Once implanted, the actuator expands into a larger electrode array beneath the skull, minimizing initial tissue disruption. Similarly, minimally invasive spinal-cord implants utilize microfluidic systems to achieve shape actuation (**Figure** **8**a).^37^ These implants validated in a cadaveric model were introduced via a small tube (<2 mm) and expanded to a width of 14 mm, comparable to common clinically available paddle electrodes.

Beyond pneumatics, a purely material-based concept can be used to yield shape changes. Nitinol, for instance, offers a promising approach (Figure 8b).^88^ This shape-memory alloy undergoes temperature-induced transformations, eliminating the need for pressure changes during deployment. This characteristic makes it particularly well suited for minimally invasive neural interfaces. Nitinol is also commonly used in endovascular stents. This concept was recently used to develop the “Stentrode,” a minimally invasive stent-mounted electrode array. Such an insertion technique avoids direct penetration and damage of the brain tissue.^89^ Histology and MRI imaging studies suggest promising biocompatibility, making it a strong candidate for chronic neural interface applications.^89^

Recent research explores electric field driven actuation as a promising approach for manipulating implant shape. This method utilizes the electrochemical swelling response of conducting polymers to induce controlled shape changes in the implant upon electrical actuation. This approach offers several potential advantages. It eliminates the need for external pressure sources (pneumatics) or bulky shape-memory alloys (nitinol), potentially enabling even smaller and more intricate implant designs.^90^

### Biointegrated

In pursuing the integration of bioelectronic devices with the body, the chemical composition of the device’s surface is crucial in modulating the FBR. Features such as hydrophobicity or hydrophilicity, surface charge, and specific immune-reactive functional groups are instrumental in guiding protein adsorption and cellular adhesion, thereby influencing the immune system’s response to the implant.^91,92^ Traditional approaches to reduce this reaction included the use of anti-inflammatory and antifibrotic agents and strategies.^93^ Examples include dexamethasone-doped electropolymerized PEDOT^94^ and the use of local delivery of inflammasome inhibitors to reduce the inflammation and fibrosis associated with neural implants.^95^ Other antifibrotic strategies include targeting colony-stimulating factor-1 receptor (CSF1R)^96^ and connective tissue growth factor (CTGF).^97^ Pro-myelinating clemastine administration improves recording performance of chronically implanted microelectrodes and nearby neuronal health.^98^

Beyond these approaches, hydrogels have emerged as a compelling solution to the challenge of biological integration, effectively addressing both mechanical and chemical disparities between the bioelectronic materials and the surrounding biological tissue. With their significantly lower stiffness relative to conventional bioelectronics materials, they offer a gentler intermediate interface with the recipient tissues.^99^ Their high water and ion content ensures an interaction that aligns closely with the body’s physiological milieu, minimizing both chemical and mechanical mismatches. The inherent hydrophilicity of hydrogels also plays a role in deterring protein adsorption, a precursor to inflammation, thus reducing the FBR and rendering them well suited for biological integration.^100^ Hydrogels have been employed to enhance the biocompatibility of thin-film electronic devices through encapsulation techniques, applicable to materials such as PEDOT:PSS and polyimide.^101^ These hydrogel-coated surfaces have been shown to diminish the adhesion and activation of inflammatory cells.^102^

**Figure 9:**
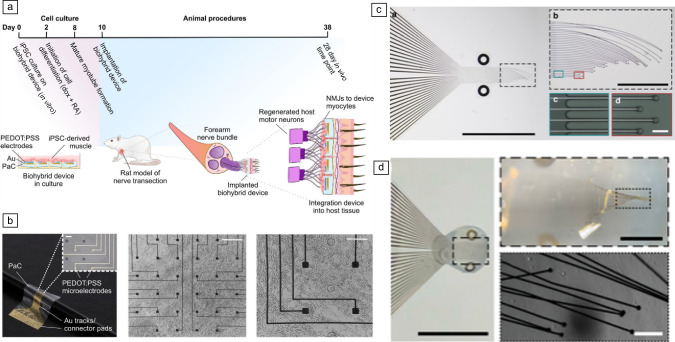
Biohybrid devices. (a) Biohybrid device for peripheral nerve repair design and fabrication. (b) Thin-film devices with cells cultured on top. Left image scale bar: 60 µm; Middle image scale bar: 465 µm; Right image scale bar: 230 µm. Reprinted with permission from Reference 103. © 2023 Wiley. (c) Construction of a hybrid implant system for creating long‐term interfaces with tissue. Left image scale bar: 5 mm; Top right image scale bar: 1 mm; Bottom right image scale bar: 50 µm. (d) Implants are created by combining a microelectrode array with a bioresorbable and remodelable collagen gel. Left image scale bar: 5 mm; Top right image scale bar: 1 mm; Bottom right image scale bar: 100 µm. Reprinted with permission from Reference 49. © 2023 Wiley. iPSC, induced pluripotent stem cell.

Building upon the concept of device integration, researchers are developing biohybrid strategies that transcend biocompatibility to achieve genuine convergence between devices and living tissues. One notable approach involves combining iPSC-derived myocytes seeded onto a fibrin hydrogel with flexible electronics (**Figure** **9**a–b).^103^ This biohybrid paradigm has successfully integrated with the peripheral nerves in animal models, establishing neuromuscular junctions and pointing toward its capability to restore function, outperforming traditional neural interfaces. Another approach utilizes bioresorbable gels combined with thin-film microelectrode arrays. These devices have shown promising long-term integration within muscle tissue in animal models, reducing scar formation and maintaining functionality (Figure 9c–d).^104^

Finally, bioresorbable electronic devices are emerging as a transformative approach in transient medical implementations, offering the distinct advantage of obviating the need for surgical retrieval.^42^ These devices, constructed with materials, such as magnesium, magnesium oxide, monocrystalline silicon nanomembranes, and silk that naturally degrade in the body, are designed for short-term therapeutic applications, dissolving after their functional period concludes.^42^ Researchers have developed ultrathin, lightweight, and multichannel neural interfaces capable of continuous high-fidelity neural signal mapping. These biocompatible interfaces degrade safely after serving their function.^105^ Further advancements in this area include on-demand bioresorbability, offering even greater control over the implant lifetime.^105^ This technology allows for the precise timing of device dissolution, triggered by specific physiological events or external stimuli, aligning therapeutic application with the exact needs of tissue healing processes or disease resolution.

## Discussion

While the potential applications and advancements in thin-film bioelectronic medicine are exciting, significant hurdles remain before widespread clinical adoption, namely (1) engineering challenges associated with miniaturized devices; (2) stability of thin-film devices and the need for standardized testing protocols; (3) integration of complete systems; (4) imaging of thin-film devices during surgical procedures; and (5) implantation and explanation of the devices.

Scaling down traditional bulky devices to the micron-sized thin-film realm presents unique challenges. The properties of thin films are different than that of bulk materials.^106^ Smaller electrodes suffer from increased edge effects, higher impedance, and require higher current densities, putting them under greater stress and at risk of electromigration, a phenomenon where electrode material is displaced due to high current flow—leading to quicker electrode degradation. Additionally, miniaturization makes these devices more susceptible to crosstalk, a form of electrical interference between neighboring electrodes.^107^ Careful consideration must be given to charge density and overall electrode effectiveness during the design process.^108^

In addition, translating these devices from research settings to real-world clinical applications necessitates demonstrating long-term safety and efficacy in pre-clinical evaluations. This, in turn, demands robust stability of the electrode.^109^ Studies have shown significant variation in electrode material degradation rates. For example, one study using an ISO-informed aging model found that gold electrodes degraded rapidly compared to conducting polymer electrodes, which lasted nearly 10× longer.^110^ This highlights the importance of material selection and the need for standardized testing methodologies to accurately assess long-term stability before clinical translation.

Developing fully integrated bioelectronic systems presents several challenges. First, managing the vast amount of data these devices collect from tissues necessitates the parallel development of robust backend infrastructure, including cabling or wireless data transmission and efficient processing methods.^111^ Multiplexing, combining signals from multiple electrodes, offers a partial solution to this data management challenge. Second, power consumption remains a significant concern. Ideally, these devices would operate wirelessly, eliminating the need for bulky implanted batteries, the current standard. Research in wireless technology and implantable battery solutions is ongoing to address this limitation.

Finally, packaging these miniaturized yet complex systems poses a challenge. Balancing soft, data-transmitting components with mechanical strength, long-term durability, and watertight seals requires innovative packaging solutions, an area of active research.^112^

In addressing the critical need for long-term or permanent implantation in chronic conditions, ensuring device compatibility with diagnostic imaging emerges as a key consideration. Moreover, the trend toward increased MRI usage in diagnostics underscores the importance of developing MRI-compatible bioelectronic devices.^113^ Electrocorticography (ECoG) grids made from soft, thin films embedded in silicone demonstrate minimal image artifacts and no adverse heating in a standard 3-T MRI scanner, highlighting the potential for safe use alongside MRI.^114^ Building upon the crucial need for MRI compatibility, another essential consideration for bioelectronic devices, especially those featuring minimally invasive insertion techniques, is their visibility under x-ray imaging. With fluoroscopy being predominant in clinical settings for device implantation and monitoring, the development of devices that are easily visualized during these processes becomes imperative.^115^ A recent study addresses this by introducing mechanically flexible x-ray markers using bismuth- and barium-infused elastomers integrated into thin-film implants.^116^ These markers do not compromise the inherent flexibility of the implants and offer the added benefit of being visible under fluoroscopy.

Clinical translation of bioelectronic devices necessitates addressing several challenges. Implantation techniques must be tailored to the delicate nature of thin-film devices, ensuring atraumatic placement without compromising functionality.^117,118^ Recent research explores degradable materials for device-shuttle connections, such as detachable 3D implanters with hydrogel-coated or degradable PEG shuttles.^119^ While promising, these methods still introduce some implantation damage, requiring further refinement. Similarly, explantation methods require careful design to minimize tissue damage during removal. Balancing electrode design remains crucial, optimizing for both flexibility (achieved by thinness) and sufficient mechanical stability for reliable implantation.

Assuming the aforementioned challenges are successfully addressed, thin-film implants present a transformative avenue for bioelectronic medicine applications. First, disease management stands to benefit greatly. As previously discussed, clinical evidence has already shown the benefits of using bioelectronic medicine in the management of epilepsy, movement disorders such as Parkinson’s disease, chronic pain, and even psychiatric disorders such as depression. These diseases are just examples of opportunities where delivering localized stimulation with minimal tissue damage opens doors for more effective and personalized treatment approaches. Second, intraoperative monitoring presents another opportunity. Surgeons could use these devices for real-time monitoring of brain, spinal cord, and nerve activity. This information is crucial for pinpointing eloquent areas during delicate procedures such as tumor removal, ultimately minimizing risks. Additionally, the high electrode density compared to traditional devices allows for comprehensive brain mapping in both clinical and research settings, leading to a deeper understanding of the nervous system. Finally, post-traumatic monitoring represents another exciting application. These devices can monitor not only neural activity but also tissues such as ligaments during post-surgical recovery, providing valuable data for optimizing rehabilitation strategies. Bioresorbable thin-film electronics are particularly promising for short-term monitoring applications. These devices naturally dissolve within the body after use, eliminating the need for explantation surgery. With ongoing human trials, thin-film bioelectronics pave the way for a transformative future in bioelectronic medicine.^118,120–122^

## Conclusion

Since the first cardiac pacemakers of the late 1950s, bioelectronic medicine has revolutionized healthcare, offering sophisticated and efficient bidirectional interaction with neural tissue and potentially even reduced long-term costs compared to traditional therapies. However, limitations exist with current manually assembled devices; the rigid electrodes can be highly invasive, requiring repeat surgeries due to micromotion-induced scarring or migration, both resulting in a substantial loss of efficacy. Additionally, the rising need for advanced interfacing necessitates electrode miniaturization. A significant shift toward microfabrication techniques has emerged to address these challenges. This technology allows for the development of thin-film electrodes, which, due to their mechanical attributes, offer superior device integration.

This article highlighted the benefits of thin-film implants for bioelectronic medicine. These advancements unlock a range of possibilities. Flexible and stretchable designs enable better apposition with tissue, reducing scarring and improving long-term functionality. Miniaturization of the electrodes leads to more precise recording and stimulation, allowing for a deeper understanding of the nervous system and the development of targeted therapies. Thin-film implants can integrate capabilities for multimodal recording and stimulation, allowing for combined therapies and gathering more comprehensive information about the body’s state. Biointegration and shape actuation features facilitate tissue repair and better integration with the body, potentially reducing the risk of rejection and improving long-term biocompatibility. Finally, minimally invasive insertion techniques made possible by flexible electrodes reduce implantation trauma and shorten recovery time.

While these thin-film bioelectronic implants offer a range of exciting possibilities, formidable challenges remain in translating them to clinical practice. Long-term stability, the design and miniaturization of closed-loop systems and their associated circuitry, compatibility with imaging techniques, packaging considerations, and the standardization of procedures are all areas requiring further development. Nevertheless, the research community is actively tackling these hurdles. This ongoing effort holds significant potential for advancing the field of bioelectronic medicine moving toward more refined and clinically translatable bioelectronic interfaces.

## Data Availability

Not applicable.

## References

[1] V.A. Pavlov, K.J. Tracey, *Neuron* **110**, 3627 (2022) 36174571 10.1016/j.neuron.2022.09.003 PMC10155266

[2] K. Famm, B. Litt, K.J. Tracey, E.S. Boyden, M. Slaoui, *Nature* **496**, 159 (2013) 23579662 10.1038/496159a PMC4179459

[3] S. Asirvatham, K. Londoner, M. Aravamudan, T. Deering, H. Heidbuchel, S. Kapa, B. Keenan, E. Maor, S. Mattke, L.T. Middleton, V. Pavlov, D. Weber, *Bioelectron. Med.* **6**, 1 (2020) 32232110 10.1186/s42234-020-0037-8 PMC7098241

[4] K.A.S. Mitchell, W. Anderson, T. Shay, J. Huang, M. Luciano, J.I. Suarez, P. Manson, H. Brem, C.R. Gordon, *Oper. Neurosurg.* **19**, 341 (2020) 10.1093/ons/opz431 PMC7594174 31993644

[5] C. Kenney, R. Simpson, C. Hunter, W. Ondo, M. Almaguer, A. Davidson, J. Jankovic, *J. Neurosurg.* **106**, 621 (2007) 17432713 10.3171/jns.2007.106.4.621

[6] M.L. Kringelbach, N. Jenkinson, S.L.F. Owen, T.Z. Aziz, *Nat. Rev. Neurosci.* **8**, 623 (2007) 17637800 10.1038/nrn2196

[7] W.C. Stacey, B. Litt, *Nat. Clin. Pract. Neurol.* **4**, 190 (2008) 18301414 10.1038/ncpneuro0750 PMC2904395

[8] L. Colloca, T. Ludman, D. Bouhassira, R. Baron, A.H. Dickenson, D. Yarnitsky, R. Freeman, A. Truini, N. Attal, N.B. Finnerup, C. Eccleston, E. Kalso, D.L. Bennett, R.H. Dworkin, S.N. Raja, *Nat. Rev. Dis. Prime.* **3**, 17002 (2017) 10.1038/nrdp.2017.2 PMC5371025 28205574

[9] D.R. Moore, R.V. Shannon, *Nat. Neurosci.* **12**, 686 (2009) 19471266 10.1038/nn.2326

[10] T. Denison, M.J. Morrell, *Neurology* **98**, 65 (2022) 35263267 10.1212/WNL.0000000000013061 PMC8762584

[11] R.H. Howland, *Curr. Behav. Neurosci. Rep.* **1**, 64 (2014) 24834378 10.1007/s40473-014-0010-5 PMC4017164

[12] A.M. Lozano, N. Lipsman, H. Bergman, P. Brown, S. Chabardes, J.W. Chang, K. Matthews, C.C. McIntyre, T.E. Schlaepfer, M. Schulder, Y. Temel, J. Volkmann, J.K. Krauss, *Nat. Rev. Neurol.* **15**, 148 (2019) 30683913 10.1038/s41582-018-0128-2 PMC6397644

[13] L.R. Hochberg, D. Bacher, B. Jarosiewicz, N.Y. Masse, J.D. Simeral, J. Vogel, S. Haddadin, J. Liu, S.S. Cash, P. Van Der Smagt, J.P. Donoghue, *Nature* **485**, 372 (2012) 22596161 10.1038/nature11076 PMC3640850

[14] C. Ethier, E.R. Oby, M.J. Bauman, L.E. Miller, *Nature* **485**, 368 (2012) 22522928 10.1038/nature10987 PMC3358575

[15] L. Brosseau, K.A. Yonge, V. Welch, S. Marchand, M. Judd, G.A. Wells, P. Tugwell, *Cochrane Database Syst. Rev.* (2003). 10.1002/14651858.CD004377/MEDIA/CDSR/CD004377/IMAGE_N/NCD004377-CMP-003-01.PNG 10.1002/14651858.CD004377 PMC8826159 12918009

[16] G. Thakral, P.J. Kim, J. LaFontaine, R. Menzies, B. Najafi, L.A. Lavery, *J. Diabetes Sci. Technol.* **7**, 1202 (2013). 10.1177/193229681300700510 24124947 10.1177/193229681300700510 PMC3876364

[17] A. Djourno, C. Eyries, *Presse Méd.* **65**, 1417 (1957) 13484817

[18] W.F. House, J. Urban, *Ann. Otol. Rhinol. Laryngol.* **82**, 504 (1973). 10.1177/000348947308200408 4721186 10.1177/000348947308200408

[19] MED-EL, How a MED-EL Cochlear Implant Is Made: Part 2. (The MED-EL Blog, 2016). https://blog.medel.com/technology/how-a-med-el-cochlear-implant-is-made-part-2/. Accessed 20 Apr 2016

[20] Cochlear^TM^, “Cochlear^TM^ Nucleus ® CI512 Physician’s Package” (2021).

[21] Boston Scientific, SCS Lead Portfolio. https://www.bostonscientific.com/en-EU/products/spinal-cord-stimulator-systems/scs_lead_portfolio.html

[22] A.L. Maldonado-Naranjo, L.A. Frizon, N.C. Sabharwal, R. Xiao, O. Hogue, D.A. Lobel, A.G. Machado, S.J. Nagel, *Neuromodulation* **21**, 513 (2018) 28833931 10.1111/ner.12643

[23] O. Veiseh, J.C. Doloff, M. Ma, A.J. Vegas, H.H. Tam, A.R. Bader, J. Li, E. Langan, J. Wyckoff, W.S. Loo, S. Jhunjhunwala, A. Chiu, S. Siebert, K. Tang, J. Hollister-Lock, S. Aresta-Dasilva, M. Bochenek, J. Mendoza-Elias, Y. Wang, M. Qi, D.M. Lavin, M. Chen, N. Dholakia, R. Thakrar, I. Lacík, G.C. Weir, J. Oberholzer, D.L. Greiner, R. Langer, D.G. Anderson, *Nat. Mater.* **14**, 643 (2015) 25985456 10.1038/nmat4290 PMC4477281

[24] M.Z. Zarzycki, I. Domitrz, *Acta Neuropsychiatr.* **32**, 57 (2020) 31452489 10.1017/neu.2019.35

[25] Activa RC® Neurostimulator for Medtronic DBS Therapy. https://www.medtronic.com/uk-en/patients/treatments-therapies/neurostimulator-essential-tremor/activa-rc.html

[26] H.H. Shen, *Proc. Natl. Acad. Sci. U.S.A.* **116**, 4764 (2019) 30862742 10.1073/pnas.1900442116 PMC6421452

[27] S.P. Lacour, G. Courtine, J. Guck, Materials and technologies for soft implantable neuroprostheses. *Nat. Rev. Mater.* **1**, 16063 (2016)

[28] J. Rivnay, H. Wang, L. Fenno, K. Deisseroth, G.G. Malliaras, *Sci. Adv.* **3**(6), e1601649 (2017). 10.1126/SCIADV.1601649/ASSET/72C9E1D9-57BC-48C4-875E-279DABCE854D/ASSETS/GRAPHIC/1601649-F8.JPEG 28630894 10.1126/sciadv.1601649 PMC5466371

[29] D. Khodagholy, J.N. Gelinas, T. Thesen, W. Doyle, O. Devinsky, G.G. Malliaras, G. Buzsáki, *Nat. Neurosci.* **18**, 310 (2015) 25531570 10.1038/nn.3905 PMC4308485

[30] J. Ordonez, M. Schuettler, C. Boehler, T. Boretius, T. Stieglitz, *MRS Bull.* **37**(6), 590 (2012)

[31] J.S. Ordonez, C. Boehler, M. Schuettler, T. Stieglitz, in *Proceedings of the Annual International Conference of the IEEE Engineering in Medicine and Biology Society* (EMBS, 2012), pp. 5134–5137 10.1109/EMBC.2012.6347149 23367084

[32] F. Ceyssens, R. Puers, *J. Neural Eng.* **12**, 054001 (2015) 26269487 10.1088/1741-2560/12/5/054001

[33] J. Pfau, D. Ganatra, A. Weltin, G. Urban, J. Kieninger, T. Stieglitz, in *Proceedings of the Annual International Conference of the IEEE Engineering in Medicine and Biology Society, EMBS* (Institute of Electrical and Electronics Engineers Inc., 2019), pp. 3762–3765 10.1109/EMBC.2019.8856621 31946693

[34] Y. Xu, C. Luo, F.G. Zeng, J.C. Middlebrooks, H.W. Lin, Z. You, *IEEE Trans. Biomed. Eng.* **66**, 573 (2019) 30004866 10.1109/TBME.2018.2850753 PMC6328340

[35] U.A. Aregueta-Robles, A.J. Woolley, L.A. Poole-Warren, N.H. Lovell, R.A. Green, *Front. Neuroeng.* **7**, 15 (2014) 24904405 10.3389/fneng.2014.00015 PMC4034607

[36] J.D. Weiland, D.J. Anderson, *IEEE Trans. Biomed. Eng.* **47**, 911 (2000) 10916262 10.1109/10.846685

[37] B.J. Woodington, V.F. Curto, Y.L. Yu, H. Martínez-Domínguez, L. Coles, G.G. Malliaras, C.M. Proctor, D.G. Barone, *Sci. Adv.* **7**, 7833 (2021) 10.1126/sciadv.abg7833 PMC8232905 34172452

[38] T. Araki, L.M. Bongartz, T. Kaiju, A. Takemoto, S. Tsuruta, T. Uemura, T. Sekitani, *Flex. Print. Electron.* **5**, 043002 (2020)

[39] D.H. Kim, J. Viventi, J.J. Amsden, J. Xiao, L. Vigeland, Y.S. Kim, J.A. Blanco, B. Panilaitis, E.S. Frechette, D. Contreras, D.L. Kaplan, F.G. Omenetto, Y. Huang, K.C. Hwang, M.R. Zakin, B. Litt, J.A. Rogers, *Nat. Mater.* **9**, 511 (2010) 20400953 10.1038/nmat2745 PMC3034223

[40] I.R. Minev, P. Musienko, A. Hirsch, Q. Barraud, N. Wenger, E.M. Moraud, J. Gandar, M. Capogrosso, T. Milekovic, L. Asboth, R.F. Torres, N. Vachicouras, Q. Liu, N. Pavlova, S. Duis, A. Larmagnac, J. Vörös, S. Micera, Z. Suo, G. Courtine, S.P. Lacour, *Science* **347**(6218), 159 (2015) 25574019 10.1126/science.1260318

[41] J. Viventi, D.H. Kim, L. Vigeland, E.S. Frechette, J.A. Blanco, Y.S. Kim, A.E. Avrin, V.R. Tiruvadi, S.W. Hwang, A.C. Vanleer, D.F. Wulsin, K. Davis, C.E. Gelber, L. Palmer, J. Van Der Spiegel, J. Wu, J. Xiao, Y. Huang, D. Contreras, J.A. Rogers, B. Litt, *Nat. Neurosci.* **14**, 1599 (2011) 22081157 10.1038/nn.2973 PMC3235709

[42] S.W. Hwang, H. Tao, D.H. Kim, H. Cheng, J.K. Song, E. Rill, M.A. Brenckle, B. Panilaitis, S.M. Won, Y.S. Kim, Y.M. Song, K.J. Yu, A.A. Ameen, R. Li, Y. Su, M. Yang, D.L. Kaplan, M.R. Zakin, M.J. Slepian, Y. Huang, F.G. Omenetto, J.A. Rogers, *Science* **337**(6102), 1640 (2012) 23019646 10.1126/science.1226325 PMC3786576

[43] S.P. Lacour, S. Benmerah, E. Tarte, J. Fitzgerald, J. Serra, S. McMahon, J. Fawcett, O. Graudejus, Z. Yu, B. Morrison, *Med. Biol. Eng. Comput.* **48**, 945 (2010) 20535574 10.1007/s11517-010-0644-8

[44] K.C. Spencer, J.C. Sy, K.B. Ramadi, A.M. Graybiel, R. Langer, M.J. Cima, *Sci. Rep.* **7**, 1 (2017) 28127051 10.1038/s41598-016-0028-x PMC5428335

[45] V.S. Polikov, P.A. Tresco, W.M. Reichert, *J. Neurosci. Methods* **148**, 1 (2005) 16198003 10.1016/j.jneumeth.2005.08.015

[46] E. Mariani, G. Lisignoli, R.M. Borzì, L. Pulsatelli, *Int. J. Mol. Sci.* **20**, 636 (2019) 30717232 10.3390/ijms20030636 PMC6386828

[47] A. Carnicer-Lombarte, D.G. Barone, I.B. Dimov, R.S. Hamilton, M. Prater, X. Zhao, A.L. Rutz, G.G. Malliaras, S.P. Lacour, C.E. Bryant, J.W. Fawcett, K. Franze, *bioRxiv* 829648 (2022)

[48] A. Carnicer-Lombarte, A.J. Boys, A. Güemes, J. Gurke, S. Velasco-Bosom, S. Hilton, D.G. Barone, G.G. Malliaras, *bioRxiv* (2023). 10.1101/2023.04.14.536862

[49] A.J. Boys, A. Carnicer-Lombarte, A. Güemes-Gonzalez, D.C. Van Niekerk, S. Hilton, D.G. Barone, C.M. Proctor, R.M. Owens, G.G. Malliaras, *Adv. Mater.* **35**, 2207847 (2023) 10.1002/adma.202207847 PMC11475589 36458737

[50] T. Boretius, T. Stieglitz, *Direct Nerve Stimulation for Induction of Sensation and Treatment of Phantom Limb Pain* (CRC Press, Boca Raton, 2019), pp. 77–133

[51] J. Badia, T. Boretius, A. Pascual-Font, E. Udina, T. Stieglitz, X. Navarro, *IEEE Trans. Biomed. Eng.* **58**, 2324 (2011) 10.1109/TBME.2011.2153850 21571604

[52] V. Paggi, F. Fallegger, L. Serex, O. Rizzo, K. Galan, A. Giannotti, I. Furfaro, C. Zinno, F. Bernini, S. Micera, S.P. Lacour, *Bioelectron. Med.* **10**, 6 (2024) 38350988 10.1186/s42234-023-00137-y PMC10865708

[53] J.F. Kurniawan, B. Tjhia, V.M. Wu, A. Shin, N.L.J. Sit, T. Pham, A. Nguyen, C. Li, R. Kumar, M. Aguilar-Rivera, I. Lerman, D.C. Kunkel, T.P. Coleman, J.F. Kurniawan, V.M. Wu, C. Li, M. Aguilar-Rivera, T.P. Coleman, B. Tjhia, A. Shin, T. Pham, R. Kumar, N.L.J. Sit, A. Nguyen, I. Lerman, D.C. Kunkel, *Adv. Mater. Technol.* **6**, 2001229 (2021)

[54] A. Sahasrabudhe, L.E. Rupprecht, S. Orguc, T. Khudiyev, T. Tanaka, J. Sands, W. Zhu, A. Tabet, M. Manthey, H. Allen, G. Loke, M.J. Antonini, D. Rosenfeld, J. Park, I.C. Garwood, W. Yan, F. Niroui, Y. Fink, A. Chandrakasan, D.V. Bohórquez, P. Anikeeva, *Nat. Biotechnol.* **42**, 892 (2024) 37349522 10.1038/s41587-023-01833-5 PMC11180606

[55] M.K. Hogan, S.M. Barber, Z. Rao, B.R. Kondiles, M. Huang, W.J. Steele, C. Yu, P.J. Horner, *Sci. Rep.* **11**, 14900 (2021) 34290260 10.1038/s41598-021-94047-1 PMC8295294

[56] D.G. Barone, B. Woodington, J. Lei, A. Carnicer-Lombarte, A. Güemes, T. Naegele, S. Hilton, S. El-Hadwe, R. Trivedi, A. Hospital, G. Malliaras, *Res. Sq.* (2023). 10.21203/rs.3.rs-3179147/v1

[57] B.D. Paulsen, K. Tybrandt, E. Stavrinidou, J. Rivnay, *Nat. Mater.* **19**, 13 (2019) 31427743 10.1038/s41563-019-0435-z

[58] M.K. Hogan, G.F. Hamilton, P.J. Horner, *Front. Cell. Neurosci.* **14**, 547839 (2020) 10.3389/fncel.2020.00271 PMC7591397 33173465

[59] S. Middya, A. Carnicer-Lombarte, V.F. Curto, A. Genewsky, A.L. Rutz, D.G. Barone, G.S.K. Schierle, A. Sirota, G. Malliaras, *SSRN Electron. J.* (2022). 10.2139/SSRN.4007579

[60] J.M. Lee, Y.W. Pyo, Y.J. Kim, J.H. Hong, Y. Jo, W. Choi, D. Lin, H.G. Park, *Nat. Commun.* **14**, 7088 (2023) 37925553 10.1038/s41467-023-42860-9 PMC10625630

[61] J. Rivnay, R.M. Owens, G.G. Malliaras, *Chem. Mater.* **26**, 679 (2014)

[62] J. Rivnay, P. Leleux, M. Ferro, M. Sessolo, A. Williamson, D.A. Koutsouras, D. Khodagholy, M. Ramuz, X. Strakosas, R.M. Owens, C. Benar, J.M. Badier, C. Bernard, G.G. Malliaras, *Sci. Adv.* **1**(4), e1400251 (2015). 10.1126/SCIADV.1400251/SUPPL_FILE/1400251_SM.PDF 26601178 10.1126/sciadv.1400251 PMC4640642

[63] E. Musk, *J. Med. Internet Res.* **21**, e16194 (2019) 31642810 10.2196/16194 PMC6914248

[64] T.M. Allen, P.R. Cullis, *Science* **303**(5665), 1818 (2004) 15031496 10.1126/science.1095833

[65] M. Mariello, E. Ismail, C.M. Proctor, *Adv. Healthc. Mater.* **2023**, 2302969 (2023) 10.1002/adhm.202302969 37924224

[66] C.M. Proctor, A. Slézia, A. Kaszas, A. Ghestem, I. del Agua, A.M. Pappa, C. Bernard, A. Williamson, G.G. Malliaras, *Sci. Adv.* **4**(8), eaau1291 (2018). 10.1126/sciadv.aau1291 30167463 10.1126/sciadv.aau1291 PMC6114990

[67] I. Cicha, R. Priefer, P. Severino, E.B. Souto, S. Jain, *Biomolecules* **12**, 1198 (2022) 36139035 10.3390/biom12091198 PMC9496590

[68] J.W. Jeong, J.G. McCall, G. Shin, Y. Zhang, R. Al-Hasani, M. Kim, S. Li, J.Y. Sim, K.I. Jang, Y. Shi, D.Y. Hong, Y. Liu, G.P. Schmitz, L. Xia, Z. He, P. Gamble, W.Z. Ray, Y. Huang, M.R. Bruchas, J.A. Rogers, *Cell* **162**, 662 (2015) 26189679 10.1016/j.cell.2015.06.058 PMC4525768

[69] C. Yu, I.R. Cassar, J. Sambangi, W.M. Grill, *J. Neurosci.* **40**, 4323 (2020) 32312888 10.1523/JNEUROSCI.3071-19.2020 PMC7252487

[70] D.W. Park, J.P. Ness, S.K. Brodnick, C. Esquibel, J. Novello, F. Atry, D.H. Baek, H. Kim, J. Bong, K.I. Swanson, A.J. Suminski, K.J. Otto, R. Pashaie, J.C. Williams, Z. Ma, *ACS Nano* **12**, 148 (2018) 29253337 10.1021/acsnano.7b04321

[71] N. Wang, A. Yang, Y. Fu, Y. Li, F. Yan, *Acc. Chem. Res.* **52**, 277 (2019) 30620566 10.1021/acs.accounts.8b00448

[72] G. Petit-Pierre, P. Colin, E. Laurer, J. Déglon, A. Bertsch, A. Thomas, B.L. Schneider, P. Renaud, *Nat. Commun.* **8**, 1239 (2017) 29093476 10.1038/s41467-017-01419-1 PMC5665973

[73] Y. Liang, T. Guo, L. Zhou, A. Offenhäusser, D. Mayer, *Materials* (Basel) **13**, 2577 (2020) 32516935 10.3390/ma13112577 PMC7321560

[74] S.L. Bidinger, S.T. Keene, S. Han, K.W. Plaxco, G.G. Malliaras, T. Hasan, *Sci. Adv.* **8**, eadd4111 (2022). 10.1126/SCIADV.ADD4111 36383656 10.1126/sciadv.add4111 PMC9668304

[75] S. Han, S. Yamamoto, A.G. Polyravas, G.G. Malliaras, S. Han, S. Yamamoto, A.G. Polyravas, G.G. Malliaras, *Adv. Mater.* **32**, 2004790 (2020) 10.1002/adma.202004790 33118196

[76] H. Tang, F. Yan, P. Lin, J. Xu, H.L.W. Chan, *Adv. Funct. Mater.* **21**, 2264 (2011)

[77] G. Scheiblin, R. Coppard, R.M. Owens, P. Mailley, G.G. Malliaras, G. Scheiblin, P. Mailley, R.M. Owens, G.G. Malliaras, R. Coppard, *Adv. Mater. Technol.* **2**, 1600141 (2017)

[78] M. Sessolo, J. Rivnay, E. Bandiello, G.G. Malliaras, H.J. Bolink, M. Sessolo, E. Bandiello, H.J. Bolink, J. Rivnay, G.G. Malliaras, *Adv. Mater.* **26**, 4803 (2014) 24862110 10.1002/adma.201400731

[79] H.H. Nguyen, S.H. Lee, U.J. Lee, C.D. Fermin, M. Kim, *Materials* (Basel) **12**, 121 (2019). 10.3390/MA12010121 30609693 10.3390/ma12010121 PMC6337536

[80] V. Naresh, N. Lee, *Sensors* (Basel) **21**, 1109 (2021) 33562639 10.3390/s21041109 PMC7915135

[81] S. Han, A.G. Polyravas, S. Wustoni, S. Inal, G.G. Malliaras, *Adv. Mater. Technol.* **6**, 2100763 (2021)

[82] K. Xie, N. Wang, X. Lin, Z. Wang, X. Zhao, P. Fang, H. Yue, J. Kim, J. Luo, S. Cui, F. Yan, P. Shi, *elife* **9**, e50345 (2020). 10.7554/ELIFE.50345 32043970 10.7554/eLife.50345 PMC7075691

[83] P.D. Donaldson, S.L. Swisher, *Phys. Status Solidi.* **219**, 2100683 (2022) 10.1002/pssa.202100683 PMC10461862 37641661

[84] S. Middya, V.F. Curto, A. Fernández-Villegas, M. Robbins, J. Gurke, E.J.M. Moonen, G.S.K. Schierle, G.G. Malliaras, *Adv. Sci.* **8**, 2004434 (2021) 10.1002/advs.202004434 PMC9539726 36246164

[85] D. Prodanov, J. Delbeke, *Front. Neurosci.* **10**, 174893 (2016) 10.3389/fnins.2016.00011 PMC4746296 26903786

[86] S. Song, F. Fallegger, A. Trouillet, K. Kim, S.P. Lacour, *Sci. Robot.* **8**(78), eadd1002 (2023). 10.1126/SCIROBOTICS.ADD1002/SUPPL_FILE/SCIROBOTICS.ADD1002_MDAR_REPRODUCIBILITY_CHECKLIST.PDF 37163609 10.1126/scirobotics.add1002

[87] S. Wei, A. Jiang, H. Sun, J. Zhu, S. Jia, X. Liu, Z. Xu, J. Zhang, Y. Shang, X. Fu, G. Li, P. Wang, Z. Xia, T. Jiang, A. Cao, X. Duan, *Nat. Commun.* **15**, 1 (2024) 38267440 10.1038/s41467-024-44805-2 PMC10808108

[88] P. Mitchell, S.C.M. Lee, P.E. Yoo, A. Morokoff, R.P. Sharma, D.L. Williams, C. Macisaac, M.E. Howard, L. Irving, I. Vrljic, C. Williams, S. Bush, A.H. Balabanski, K.J. Drummond, P. Desmond, D. Weber, T. Denison, S. Mathers, T.J. O’brien, J. Mocco, D.B. Grayden, D.S. Liebeskind, N.L. Opie, T.J. Oxley, B.C.V. Campbell, *JAMA Neurol.* **80**, 270 (2023) 36622685 10.1001/jamaneurol.2022.4847 PMC9857731

[89] C. Dong, A. Carnicer-Lombarte, F. Bonafè, B. Huang, S. Middya, A. Jin, X. Tao, S. Han, M. Bance, D.G. Barone, B. Fraboni, G.G. Malliaras, *Nat. Mater.* **23**, 969 (2024) 38671159 10.1038/s41563-024-01886-0 PMC11230894

[90] A.J. Vegas, O. Veiseh, J.C. Doloff, M. Ma, H.H. Tam, K. Bratlie, J. Li, A.R. Bader, E. Langan, K. Olejnik, P. Fenton, J.W. Kang, J. Hollister-Locke, M.A. Bochenek, A. Chiu, S. Siebert, K. Tang, S. Jhunjhunwala, S. Aresta-Dasilva, N. Dholakia, R. Thakrar, T. Vietti, M. Chen, J. Cohen, K. Siniakowicz, M. Qi, J. McGarrigle, S. Lyle, D.M. Harlan, D.L. Greiner, J. Oberholzer, G.C. Weir, R. Langer, D.G. Anderson, *Nat. Biotechnol.* **34**, 345 (2016) 26807527 10.1038/nbt.3462 PMC4904301

[91] A.E. Rochford, A. Carnicer-Lombarte, V.F. Curto, G.G. Malliaras, D.G. Barone, *Adv. Mater.* **32**, 1903182 (2020) 10.1002/adma.201903182 31517403

[92] Y. Zhong, R.V. Bellamkonda, *Brain Res.* **1148**, 15 (2007) 17376408 10.1016/j.brainres.2007.02.024 PMC1950487

[93] N.A. Alba, Z.J. Du, K.A. Catt, T.D.Y. Kozai, X.T. Cui, *Biosensors* (Basel) **5**, 618 (2015) 26473938 10.3390/bios5040618 PMC4697137

[94] D.G. Barone, A. Carnicer-Lombarte, P. Tourlomousis, R.S. Hamilton, M. Prater, A.L. Rutz, I.B. Dimov, G.G. Malliaras, S.P. Lacour, A.A.B. Robertson, K. Franze, J.W. Fawcett, C.E. Bryant, *Proc. Natl. Acad. Sci. U.S.A.* **119**, e2115857119 (2022) 35298334 10.1073/pnas.2115857119 PMC8944905

[95] J.C. Doloff, O. Veiseh, A.J. Vegas, H.H. Tam, S. Farah, M. Ma, J. Li, A. Bader, A. Chiu, A. Sadraei, S. Aresta-Dasilva, M. Griffin, S. Jhunjhunwala, M. Webber, S. Siebert, K. Tang, M. Chen, E. Langan, N. Dholokia, R. Thakrar, M. Qi, J. Oberholzer, D.L. Greiner, R. Langer, D.G. Anderson, *Nat. Mater.* **16**, 671 (2017) 28319612 10.1038/nmat4866 PMC5445003

[96] B. Ji, Z. Guo, M. Wang, B. Yang, X. Wang, W. Li, J. Liu, *Microsyst. Nanoeng.* **4**, 27 (2018) 31057915 10.1038/s41378-018-0027-0 PMC6220173

[97] K. Chen, F. Cambi, T.D.Y. Kozai, *Biomaterials* **301**, 122210 (2023) 37413842 10.1016/j.biomaterials.2023.122210 PMC10528716

[98] S.H. Sunwoo, S.I. Han, H. Joo, G.D. Cha, D. Kim, S.H. Choi, T. Hyeon, D.H. Kim, *Matter* **3**, 1923 (2020)

[99] M. Gori, G. Vadalà, S.M. Giannitelli, V. Denaro, G. Di Pino, *Front. Bioeng. Biotechnol.* **9**, 659033 (2021) 34113605 10.3389/fbioe.2021.659033 PMC8185207

[100] S. Lee, K. Park, J. Kum, S. An, K.J. Yu, H. Kim, M. Shin, D. Son, *Polymers* **15**, 84 (2022) 36616434 10.3390/polym15010084 PMC9824691

[101] M. Gori, S.M. Giannitelli, G. Vadalà, R. Papalia, L. Zollo, M. Sanchez, M. Trombetta, A. Rainer, G. Di Pino, V. Denaro, *Molecules* **27**, 3126 (2022) 35630604 10.3390/molecules27103126 PMC9147366

[102] A.E. Rochford, A. Carnicer-Lombarte, M. Kawan, A. Jin, S. Hilton, V.F. Curto, A.L. Rutz, T. Moreau, M.R.N. Kotter, G.G. Malliaras, D.G. Barone, *Sci. Adv.* **9**(12), eadd8162 (2023). 10.1126/SCIADV.ADD8162/SUPPL_FILE/SCIADV.ADD8162_SM.PDF 36947608 10.1126/sciadv.add8162 PMC10032597

[103] A.J. Boys, A. Carnicer-Lombarte, A. Güemes-Gonzalez, D.C. Van Niekerk, S. Hilton, D.G. Barone, C.M. Proctor, R.M. Owens, G.G. Malliaras, A.J. Boys, D.C. Van Niekerk, R.M. Owens, A. Carnicer-Lombarte, A. Güemes-Gonzalez, S. Hilton, C.M. Proctor, G.G. Malliaras, D.G. Barone, *Adv. Mater.* **35**, 2207847 (2023) 10.1002/adma.202207847 PMC11475589 36458737

[104] M. Wu, K. Yao, N. Huang, H. Li, J. Zhou, R. Shi, J. Li, X. Huang, J. Li, H. Jia, Z. Gao, T.H. Wong, D. Li, S. Hou, Y. Liu, S. Zhang, E. Song, J. Yu, X. Yu, *Adv. Sci.* **10**, 2300504 (2023) 10.1002/advs.202300504 PMC10190644 36825679

[105] M. Ohring, *Laser-Induced Damage in Optical Materials: 1993* (SPIE, 1994), p. 624

[106] Y. Qiang, W. Gu, Z. Liu, S. Liang, J.H. Ryu, K.J. Seo, W. Liu, H. Fang, *Nano Res.* **14**, 3240 (2021) 34394850 10.1007/s12274-021-3442-8 PMC8361849

[107] E.N. Nicolai, J. Arturo Larco, S.I. Madhani, N. Lan, M. Daroux, J. Thomas Mortimer, Y. Nakanishi, W. Sriitsaranusorn, G. Schiavone, N. Vachicouras, Y. Vyza, S.P. Lacour, *J. Neural Eng.* **18**, 046054 (2021) 10.1088/1741-2552/abf607 33831857

[108] P. Oldroyd, G.G. Malliaras, *Acta Biomater.* **139**, 65 (2022) 34020055 10.1016/j.actbio.2021.05.004

[109] P. Oldroyd, J. Gurke, G.G. Malliaras, P. Oldroyd, J. Gurke, G.G. Malliaras, *Adv. Funct. Mater.* **33**, 2208881 (2023)

[110] I. Rachinskiy, L. Wong, C.H. Chiang, C. Wang, M. Trumpis, J.I. Ogren, Z. Hu, B. McLaughlin, J. Viventi, *Front. Nanotechnol.* **4**, 837328 (2022) 35898702 10.3389/fnano.2022.837328 PMC9310058

[111] F. Fallegger, A. Trouillet, F.V. Coen, G. Schiavone, S.P. Lacour, *APL Bioeng.* **7**, 36109 (2023) 10.1063/5.0152509 PMC10439817 37600068

[112] R. Smith-Bindman, M.L. Kwan, E.C. Marlow, M.K. Theis, W. Bolch, S.Y. Cheng, E.J.A. Bowles, J.R. Duncan, R.T. Greenlee, L.H. Kushi, J.D. Pole, A.K. Rahm, N.K. Stout, S. Weinmann, D.L. Miglioretti, *JAMA* **322**, 843 (2019) 31479136 10.1001/jama.2019.11456 PMC6724186

[113] F. Fallegger, G. Schiavone, E. Pirondini, F.B. Wagner, N. Vachicouras, L. Serex, G. Zegarek, A. May, P. Constanthin, M. Palma, M. Khoshnevis, D. Van Roost, B. Yvert, G. Courtine, K. Schaller, J. Bloch, S.P. Lacour, *Adv. Sci.* **8**, 2003761 (2021) 10.1002/advs.202003761 PMC8097365 33977054

[114] N.E. Shalom, G.X. Gong, M. Auster, *World J. Radiol.* **12**, 213 (2020) 33240462 10.4329/wjr.v12.i10.213 PMC7653184

[115] B.J. Woodington, L. Coles, A.E. Rochford, P. Freeman, S. Sawiak, S.J.K. O’neill, O.A. Scherman, D.G. Barone, C.M. Proctor, G.G. Malliaras, B.J. Woodington, L. Coles, A.E. Rochford, D.G. Barone, C.M. Proctor, G.G. Malliaras, P. Freeman, S. Sawiak, S.J.K. O’neill, O.A. Scherman, *Adv. Healthc. Mater.* **11**, 2200739 (2022) 10.1002/adhm.202200739 PMC11468128 35871265

[116] P. Cvančara, T. Boretius, V.M. López Lvarez, P. Maciejasz, D. Andreu, S. Raspopovic, F. Petrini, S. Micera, G. Granata, E. Fernandez, P.M. Rossini, K. Yoshida, W. Jensen, J.L. Divoux, D. Guiraud, X. Navarro, T. Stieglitz, *J. Neural Eng.* **17**, 46006 (2020) 10.1088/1741-2552/ab9a9a 32512544

[117] K. Woeppel, C. Hughes, A.J. Herrera, J.R. Eles, E.C. Tyler-Kabara, R.A. Gaunt, J.L. Collinger, X.T. Cui, *Front. Bioeng. Biotechnol.* **9**, 759711 (2021) 34950640 10.3389/fbioe.2021.759711 PMC8688945

[118] S. Middya, A. Carnicer-Lombarte, V.F. Curto, S. Hilton, A. Genewsky, A.L. Rutz, D.G. Barone, G.S. Kaminski Schierle, A. Sirota, G.G. Malliaras, *Adv. Electron Mater.* **9**, 2200883 (2023)

[119] D. Khodagholy, J.N. Gelinas, Z. Zhao, M. Yeh, M. Long, J.D. Greenlee, W. Doyle, O. Devinsky, G. Buzsáki, *Sci. Adv.* **2**(11), e1601027 (2016). 10.1126/SCIADV.1601027/SUPPL_FILE/1601027_SM.PDF 28861464 10.1126/sciadv.1601027 PMC5569954

[120] B. Coughlin, W. Muñoz, Y. Kfir, M.J. Young, D. Meszéna, M. Jamali, I. Caprara, R. Hardstone, A. Khanna, M.L. Mustroph, E.M. Trautmann, C. Windolf, E. Varol, D.J. Soper, S.D. Stavisky, M. Welkenhuysen, B. Dutta, K.V. Shenoy, L.R. Hochberg, R. Mark Richardson, Z.M. Williams, S.S. Cash, A.C. Paulk, *Nat. Protoc.* **18**, 2927 (2023) 37697108 10.1038/s41596-023-00871-2

[121] K. Lee, A.C. Paulk, Y.G. Ro, D.R. Cleary, K.J. Tonsfeldt, Y. Kfir, J.S. Pezaris, Y. Tchoe, J. Lee, A.M. Bourhis, R. Vatsyayan, J.R. Martin, S.M. Russman, J.C. Yang, A. Baohan, R.M. Richardson, Z.M. Williams, S.I. Fried, U. Hoi Sang, A.M. Raslan, S. Ben-Haim, E. Halgren, S.S. Cash, S.A. Dayeh, *Nat. Commun.* **15**, 218 (2024) 38233418 10.1038/s41467-023-43727-9 PMC10794240

[122] D.D. Zhou, J.D. Dorn, R.J. Greenberg, in *Electronic Proceedings of the 2013 IEEE International Conference on Multimedia and Expo Workshops, ICMEW 2013* (2013). 10.1109/ICMEW.2013.6618428

